# Hyaluronic and Succinic Acid: New Biostimulating Combination to Counteract Dermal and Subcutaneous Aging

**DOI:** 10.3390/ijms26157548

**Published:** 2025-08-05

**Authors:** Alfredo Martinez-Gutierrez, Helena Cami, Teresa Noya, Susana Gómez-Escalante, Aina Miró Llosas, Mari Carmen González

**Affiliations:** 1 Biotechnology Unit, Mesoestetic Pharma Group, 08840 Barcelona, Spain; hcami@mesoestetic.com (H.C.); tnoya@mesoestetic.com (T.N.); 2 Medical Affairs, Mesoestetic Pharma Group, 08840 Barcelona, Spain; sgomez@mesoestetic.com (S.G.-E.); amiro@mesoestetic.com (A.M.L.); 3 R+D Department, Mesoestetic Pharma Group, 08840 Barcelona, Spain; mcgonzalez@mesoestetic.com

**Keywords:** skin aging, fibroblast senescence, dermal filler, mitophagy, adipocyte aging, biostimulation

## Abstract

Various biomaterials are currently employed for dermal biostimulation and filling purposes, with hyaluronic acid (HA)-based fillers among those with the most favorable safety profile, albeit exhibiting a limited biostimulatory effect. This study suggests that hyaluronic acid and succinic acid (SA) can significantly induce beneficial effects on skin cells by targeting key aging hallmarks. Human dermal senescent fibroblasts and aged adipocytes were treated with HA + SA, and various aging characteristics were examined through gene expression analysis and microscopy staining. HA was found to stimulate autophagy gene expression, while SA modulated senescence-gene expression, and the combination of these compounds induced mitophagy in senescent fibroblasts. Additionally, the HA + SA promoted adipogenesis, increased *IGF1*, and decreased *TNFA* gene expression in aged adipocytes. Furthermore, the conditioned medium from adipocytes treated with HA + SA upregulated key dermal genes such as *COL3A1* and *EGF*. The findings of this study suggest that HA and SA compounds can be used for the biostimulation of aged skin through the regulation of senescence-associated gene expression and cell communication between dermal fibroblasts and subcutaneous adipocytes.

## 1. Introduction

Skin aging is a complex biological process characterized by the gradual decline in skin structure and function, leading to visible signs such as wrinkles, loss of elasticity, and pigmentation changes. Among the different treatment options for these alterations, injectable biostimulators have become increasingly popular in aesthetic medicine for their ability to rejuvenate and enhance facial appearance. These treatments include biocompatible and biodegradable materials that aim to address age-related changes in the skin, such as volume loss, wrinkles, and decreased elasticity.

Different biomaterials are available in the market to counteract these skin changes. On one hand, hyaluronic acid (HA)-based fillers are typically used for restoring volume, show a better safety profile, and are claimed to exert more limited/delayed biostimulation of cells [1,2]. Most of the observed benefits of HA are mediated by its binding to cellular receptors such as CD44 and providing a 3D scaffold that enables mechanical stretching of dermal cells to effectively perform their physiological roles. Both mechanisms activate the endogenous pathways of dermal fibroblasts to enhance extracellular matrix production and regenerative responses. On the other hand, synthetic polymers such as polylactic acid (PLA) or calcium hydroxyapatite (CaHA) aim at improving skin quality without increasing skin volume while possessing more adverse effects. This lower safety profile is given by the intrinsic mechanism of action of these materials, which is based on inducing a controlled inflammatory reaction to a foreign body. This inflammatory insult activates dermal fibroblasts, which end up producing more collagen deposition [3,4].

These dermal fillers are aimed at different skin layers, mainly the dermis and hypodermis. In the dermis, the goal is to stimulate fibroblasts to produce more collagen and other extracellular matrix proteins that provide structural support. At the cellular level, these fibroblasts present most of the widely defined hallmarks of aging, including genomic instability, disabled autophagy, cellular senescence, altered cell communication, and mitochondrial dysfunction [5]. On the other hand, targeting the hypodermis aims at redensifying and restoring the volume loss caused by decreased fat accumulation. These changes are associated with the decreased adipogenic capacity of aged adipocytes and increased local inflammation [6,7].

In this skin environment context, dermal and hypodermal aging translates into decreased extracellular matrix components, inflammaging, and consequently loss of volume, firmness, and elasticity. In the dermis, fibroblasts exhibit a senescence-associated secretory phenotype (SASP), characterized by a cessation of proliferation while remaining metabolically active. These cells secrete proinflammatory cytokines, such as interleukins (ILs), and matrix metalloproteinases (MMPs), thereby contributing to the process of inflammaging [8]. Thus, new injectables that produce more natural changes, with a higher biostimulatory effect, while keeping a good safety profile, are increasingly needed. Bioactive ingredients such as endogenous compounds could fill this gap through the regulation of skin aging changes at the molecular and cellular level, both in dermal fibroblasts and subcutaneous adipocytes. Here we hypothesized that combining HA with bioactive endogenous compounds such as succinic acid (SA) could trigger a beneficial response in skin through the regulation of skin aging hallmarks. The combination of HA and SA positively regulated senescence features in dermal fibroblasts and induced adipogenesis and extracellular matrix-stimulating factors in aged adipocytes.

## 2. Results

### 2.1. Effect of Hyaluronic Acid and Succinic Acid on Human Dermal Senescent Fibroblasts

To determine the effect of both compounds on dermal aging, human dermal senescent fibroblasts were treated with either compound for 48 h. Before treatment with the compounds, the senescent state was successfully induced as observed by increased SA-b-gal staining and *CDKN2A* upregulation (Supplementary Figure S1). After the treatment, qPCR analysis revealed that hyaluronic acid had limited effects on senescence and extracellular matrix genes, while it showed a significant increase in autophagy-related genes (Table 1). On the other hand, succinic acid significantly upregulated extracellular matrix-related genes, including *COL1A1*, *CTGF*, *FBN1*, *TGF-β1*, and *LOXL1*, while it downregulated genes involved in the senescent-associated secretory phenotype including *MMP1*, *MMP3*, *CXCL10*, *IL-6*, and *IL-8* (Table 1). Thus, succinic acid induced a clear benefit in senescence-associated gene expression by regulating some age and senescent-related genes on dermal fibroblasts. Interestingly, succinic acid also upregulated *PGC1A*, a master regulator of mitochondrial biogenesis and energy metabolism [9].

**Figure 1:**
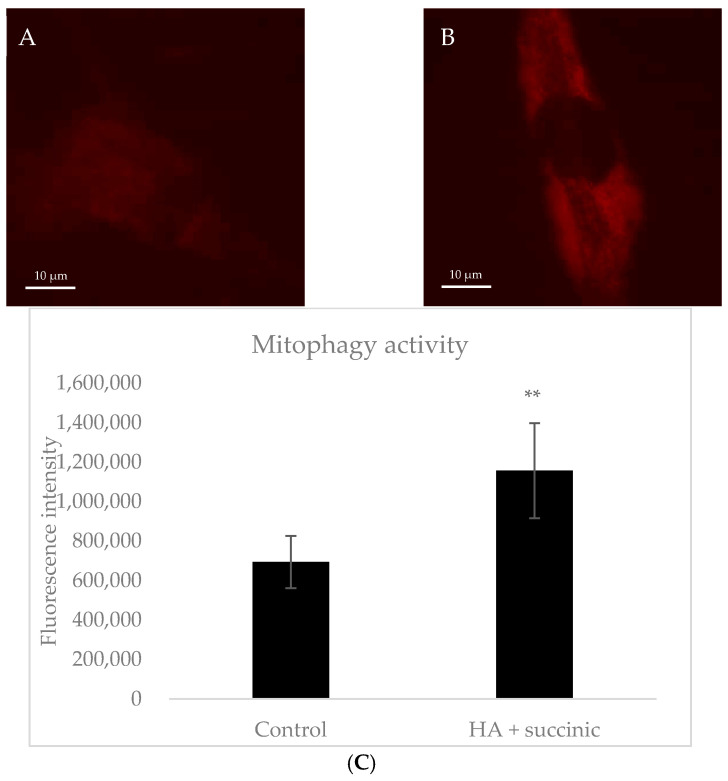
Representative fluorescent microscopy images of control senescent fibroblasts (**A**) and senescent fibroblasts treated with hyaluronic acid plus succinic acid (**B**) for 48 h using the Mtphagy dye. Red fluorescent intensity corresponds to increased mitochondria degradation in lysosomes (mitophagic activity). Scale bar: 10 µm. (**C**) Mitophagy activity of the corresponding conditions based on fluorescence intensity quantified using ImageJ software. Statistical analysis is performed using Student’s *t* test, where ** stands for *p* < 0.01.

Given that hyaluronic acid and succinic acid positively regulated autophagy and mitochondria biogenesis genes, respectively, we hypothesized that the combination of both compounds could activate the mitophagy pathway, which is based on the selective degradation of damaged mitochondria as a homeostatic process to maintain proper cellular health and functions [10]. For this, we treated dermal senescent fibroblasts with hyaluronic acid plus succinic acid and studied mitophagy activation through the fluorescent dye Mtphagy. This dye accumulated in intact mitochondria and exhibits low fluorescence under physiological conditions. When mitophagy is activated, mitochondria fuse to the lysosomes and emit high fluorescence caused by the acidic pH. As observed in Figure 1, hyaluronic acid plus succinic acid-treated cells showed higher fluorescent intensity levels, indicating that the compounds induced mitophagy in dermal senescent fibroblasts.

### 2.2. Effect of Hyaluronic Acid and Succinic Acid on Human Subcutaneous Adipocytes

**Figure 2:**
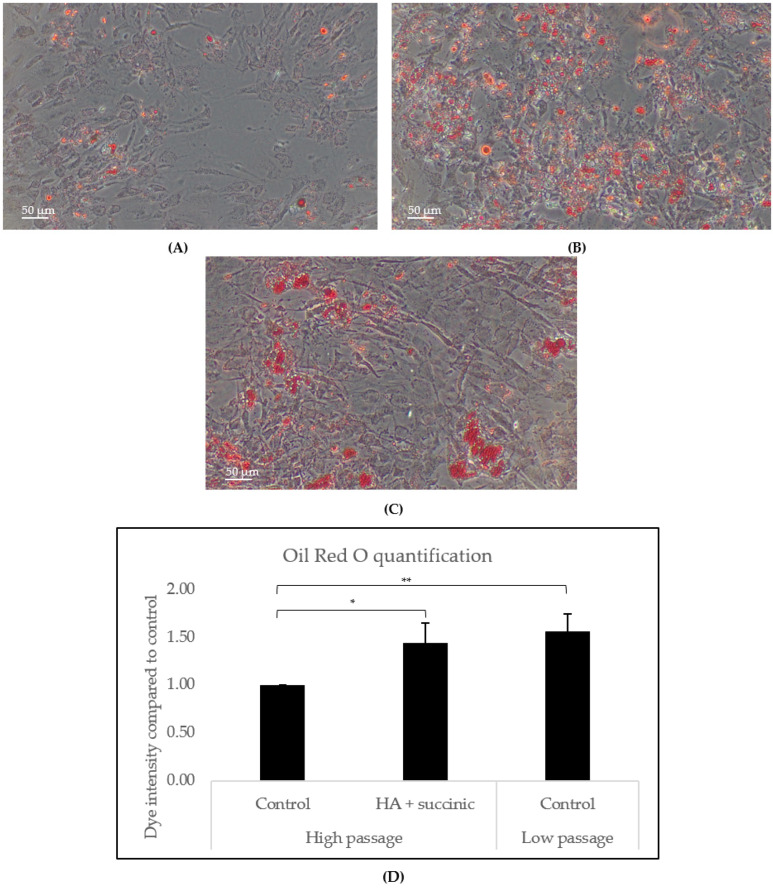
Oil red O staining of high-passage (passage 7–8) subcutaneous preadipocytes after 17 days of differentiation and treatment without (**A**) or with (**B**) hyaluronic acid plus succinic acid. Low-passage (passage 1–2) subcutaneous preadipocytes are used as a positive control of physiological adipogenesis (**C**). Scale bar: 50 µm. (**D**) Oil red O intensity quantification for the corresponding conditions. Statistical analysis is performed using Student’s *t* test, where * stands for *p* < 0.05 and ** stands for *p* < 0.01.

As subcutaneous loss of fat also contributes to facial aging features, we also studied the effect of hyaluronic acid and succinic acid on subcutaneous adipocytes. Human subcutaneous adipocytes were differentiated according to the manufacturer’s instructions and treated with compounds for 17 days. Oil red O staining was then performed to stain intracellular lipids. Aged adipocytes (high-passage) treated with the combination of compounds accumulated more fat (Figure 2B) compared to aged control adipocytes (Figure 2A), indicating that hyaluronic acid and succinic acid boost adipogenesis in aged cells from the hypodermis. Low-passage adipocytes were used as a control for normal adipogenesis induction.

**Figure 3:**
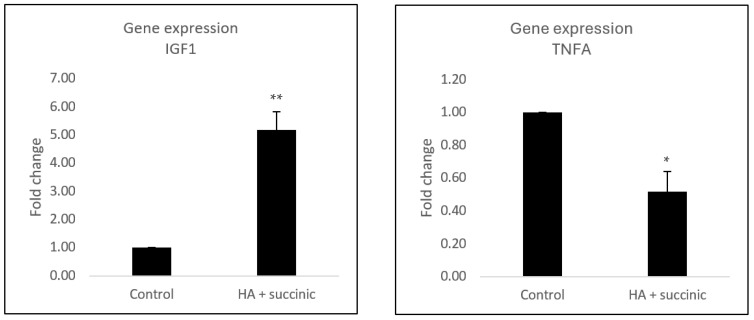
Gene expression of *IGF1* and *TNFA* in high-passage (passage 7–8) subcutaneous preadipocytes after 17 days of differentiation and treatment with or without hyaluronic acid plus succinic acid. Results are expressed in fold change compared to untreated control. Bar graphs indicate mean ± SD (*n* = 3), and Student’s *t*-test for statistical analysis represents * for *p* < 0.05 and ** for *p* < 0.01.

In parallel, aged adipocytes treated with the compounds were harvested to study the effect of hyaluronic acid and succinic acid on adipogenic and local inflammatory markers (Figure 3). This assay showed that the combination upregulated the levels of *IGF1*, a growth factor with adipogenic effects [11], while it downregulated the levels of *TNFA*, a proinflammatory cytokine that contributes to local inflammatory damage and impairs proper adipocyte functions [7].

### 2.3. Effect of Hyaluronic Acid and Succinic Acid on Human Subcutaneous Adipocytes and Dermal Fibroblast Communication

Previous research has shown that cytokines and growth factors secreted by subcutaneous adipocytes can regulate the activity of fibroblasts located in the dermis [12,13]. Given that hyaluronic acid and/or succinic acid can upregulate the levels of factors such as IGF1 in subcutaneous adipocytes (Figure 3), we hypothesized that these compounds could stimulate dermal fibroblasts through their positive effect on subcutaneous adipocytes, thereby regulating the communication between both cell types.

To prove this, we harvested the cell culture medium from aged adipocytes treated with hyaluronic acid plus succinic acid and incubated dermal fibroblasts for 24 h with this medium (conditioned medium). Dermal fibroblasts were then harvested, and extracellular matrix and growth factor genes were evaluated through qPCR analysis. As shown in Table 2, some genes were altered in fibroblasts treated with aged adipocyte-conditioned medium. Interestingly, genes such as *ELN*, *EGF*, *FGFb*, and *TGFb1* were downregulated in dermal fibroblasts, meaning that the factors secreted by aged adipocytes have a negative impact on dermal fibroblast function and extracellular protein synthesis. In contrast, age adipocyte-conditioned medium from adipocytes treated with hyaluronic acid plus succinic acid significantly upregulated *COL3A1*, *FN1*, *EGF*, and *ACTA2*, indicating that these compounds can restore and positively impact dermal fibroblast function through secreted factors from aged adipocytes.

## 3. Discussion

Skin aging is a complex biological process characterized by the progressive decline of structural, functional, and regenerative capacity in dermal and subcutaneous compartments. Hallmark features include extracellular matrix degradation, fibroblast senescence, chronic low-grade inflammation, and adipose tissue atrophy [14,15]. In aesthetic medicine, hyaluronic acid (HA)-based dermal fillers have become a cornerstone in the management of facial aging, primarily due to their viscoelastic properties and capacity to restore volume and hydration. Alternative biomaterials, including calcium hydroxyapatite and polylactic acid, are likewise employed to stimulate bioactivity in dermal cells, thereby promoting collagen synthesis and ameliorating the manifestations of skin aging; however, these materials are associated with a greater incidence of adverse effects compared to hyaluronic acid [2]. Regarding the biostimulatory effects of HA, studies have shown that multiple sessions of HA treatments are needed to restore the extracellular matrix in an efficient way [1,16]. Within this framework, there is an escalating demand for innovative dermal fillers that exhibit high efficacy while maintaining a commendable safety profile. Here, the present study offers new data on how HA and SA can positively regulate some features of aged dermal fibroblasts and subcutaneous adipocytes.

First, we explored the biological effects of HA and SA in dermal senescent fibroblasts. HA alone induced autophagy-related gene expression in senescent fibroblasts, while succinic acid modulated the senescence-associated secretory phenotype (SASP), upregulating pro-regenerative markers (e.g., *COL1A1*, *CTGF*, and *TGF-β1*) and downregulating catabolic and inflammatory mediators (*MMP1*, *CXCL10*, and *IL-6*). Previous research indicated that HA stimulates certain intracellular pathways in dermal fibroblasts, mainly through specific receptors such as CD44, which ultimately increase fibroblast proliferation [17]. Specifically, high-molecular-weight hyaluronic acid induces CD44 receptor clustering on the cell membrane, triggering downstream signaling pathways involved in proliferation, migration, and inflammation, among others [18,19]. However, to our knowledge, the effect of HA on autophagy genes in senescent fibroblasts has not been described before, which sheds more light on the mechanism of action of this filler in dermal cells. Intriguingly, CD44 has been described to regulate some of the genes that succinic acid positively regulates according to our experiment (such as TGF-*β*1 or COL1A1) [20,21], which suggests that succinic acid and hyaluronic acid could be synergistically acting through CD44 signaling to regulate dermal fibroblast function. Thus, further research aimed at unveiling the effect of this combination of compounds on CD44 signaling is warranted.

On the other hand, succinic acid acts as an intermediate in cell metabolic pathways, and as such it activates mitochondrial respiration to produce cell energy [22]. It also exerts antioxidant effects by reducing lipid peroxidation [23]. Interestingly, previous research claimed that succinic acid could also exert senescence-preventing effects after the treatment of cells during many passages and under hypoxic conditions [9]. Finally, regarding inflammation regulation, succinic acid can act both as a proinflammatory as well as an inflammation resolution factor depending on the context [24]. Here, we showed that succinic acid induces positive effects on dermal senescent fibroblasts by boosting the activation of extracellular matrix genes and downregulating the gene expression of senescent-associated secretory phenotype (SASP) genes. Thus, these results add novel contributions to the already known mechanism of action of succinic acid in the skin environment.

Based on the effect of HA and SA on autophagy and mitochondria biogenesis, respectively (Table 1), our next experiment focused on elucidating the effect of the combination of HA and SA on mitophagy. This process is defined as the recycling of damaged mitochondria through autophagy, being a key cellular pathway to respond against stress to restore mitochondrial functions in aged cells [10]. As such, it is also included in the list of hallmarks of aging [5], which highlights its importance in proper cell functions. As shown in our results, the combination of compounds effectively stimulated mitophagy in dermal senescent fibroblasts, thereby inducing the repair of damaged mitochondria. This effect could be explained by the individual effects of HA and SA on stimulating autophagy and mitochondrial biogenesis.

Considering that injectable fillers can be injected beyond the dermis [4], we aimed to investigate the effect of HA and SA on subcutaneous preadipocytes, which represent the most predominant cell within the hypodermis. The rationale of targeting this layer with injectable fillers lies in trying to counteract the loss of subcutaneous fat that occurs in aging, mainly in the facial region. Previous research showed that common features of aged subcutaneous adipose tissue are reduced adipogenic capacity of preadipocyte cells and increased local inflammation levels [7], which, at the same time, fed the reduced adipogenic activation. Here, we showed that the combination of HA and SA stimulates adipogenesis in aged preadipocytes (Figure 2). Our gene expression analysis revealed that HA + SA upregulated the levels of *IGF1* while reducing the levels of *TNFa*. These changes could explain the observed adipogenic effect of HA and/or SA, given that IGF1 is a growth factor that induces adipogenesis in the subcutaneous tissue, while TNFα is antiadipogenic and lipolytic [7,11]. These results indicate that this combination of compounds contributes to the restoration of adipose tissue homeostasis, which could translate into a redensification effect to mitigate the firmness loss and wrinkle appearance in facial aged skin. Remarkably, this proadipogenic effect has further applications beyond aging skin, such as the recent description of rapid subcutaneous fat reduction caused by weight loss therapies such as GLP1 analogs, a condition defined as “ozempic face” [25,26].

Perhaps most notably, our last experiment aimed at characterizing how the positive effect of HA and SA on aged adipocytes could affect human dermal fibroblasts through paracrine factors. Previous research has shown that secreted proteins by subcutaneous adipocytes can reach the dermis and regulate dermal fibroblasts’ function [12]. Interestingly, our results show that aged adipocyte-conditioned medium can alter the gene expression of key extracellular matrix and growth factor genes in human dermal fibroblasts, including *ELN, EGF, FGFb*, or *TGFb1* (Table 2). This highlights the key role of subcutaneous tissue beyond the commonly used direct stimulation of dermal fibroblasts to counteract the skin aging signs attributed to loss of collagen and other extracellular matrix proteins. However, as adipocyte-conditioned medium control from healthy cells was not tested, we cannot determine if this detrimental effect is caused by the factors released by aged adipocytes or factors that are initially present as medium components. Further experiments including this control condition might shed light on the specific contribution of factors produced by aged adipocytes and the components that are part of the medium formula. Regarding the treatment with the combination of compounds, HA + SA could upregulate the levels of several of these markers, including *COL3A1, FN1, EGF* and *ACTA2,* shifting fibroblast expression profiles from a senescent to a more regenerative phenotype. Noteworthy, ACTA2 is a common marker used to identify myofibroblasts, which are the activated form of fibroblasts that produce higher levels of extracellular matrix proteins and growth factors and are reduced in aged skin [27]. In addition, the fact that other genes including *COL1A1*, *ELN*, or *TGFb1* were not upregulated by the compounds suggests that the observed beneficial effects on the rest of the tested markers might be TGFb1-independent in these cells.

Finally, our results show interesting effects of HA and SA on aged fibroblasts and adipocytes. However, the individual contribution of these ingredients in dermal fibroblast mitophagy and subcutaneous aged adipocyte features remains unexplored. Thus, future experiments that describe the individual effect of HA and SA on these pathways are warranted.

Our findings advocate for a paradigm shift in how we view dermal fillers. Instead of merely serving as volumizing agents, HA-based fillers combined with bioactive compounds such as SA could also be used as skin biostimulators by targeting the key hallmarks of aging not only at the dermal level but also at the subcutaneous level. This complete approach implies a significant improvement in HA-based filler efficacy by improving the biological health of aging skin. Future clinical studies proving the effect of this combination of compounds are essential to explore the full potential of these bioactive fillers.

## 4. Materials and Methods

### 4.1. Cell Culture

Human dermal adult fibroblasts (Promocell, Heidelberg, Germany) were cultured in DMEM (Capricorn Scientific GmbH, Ebsdorfergrund, Germany) supplemented with 10% fetal bovine serum (FBS) (Fisher Scientific, Hampton, NH, USA) and Zellshield (Minerva Biolabs GmbH, Berlin, Germany). Human subcutaneous preadipocytes (Promocell, Heidelberg, Germany) were cultured in preadipocyte culture medium supplemented with Zellshield (Minerva Biolabs GmbH, Berlin, Germany). Both cell types were incubated at 37 °C in 5% CO_2_. Unless specified, all reagents were obtained from Merck Life Science, Darmstadt, Germany.

High-molecular-weight hyaluronic acid (>1000 KDa) and succinic acid treatment concentrations used for the different assays were 0.1% and 0.01%, respectively, based on previous research [28,29].

### 4.2. Gene Expression Assay in Fibroblast Dermal Senescent Cells

To study the effect of the compounds on senescent fibroblasts, cellular senescence was first induced by repetitive UVB damage. For this, human dermal fibroblasts were irradiated with 25 mJ/cm^2^ of UVB light using a Bio-Link Crosslinker BLX-E365 device (Vilber Lourmat, Marne-la-Vallée, France), incubated for 48 h, irradiated again with 25 mJ/cm^2^ of UVB light, and incubated for 72 h more. Senescence induction was confirmed by performing senescence-associated beta-galactosidase (SA-b-gal) staining and p16 (CDKN2A) gene expression as previously described [30]. Cells were then treated with 0.1% hyaluronic acid or 0.01% succinic acid for 48 h and harvested for gene expression analysis. Total RNA was extracted using the Total RNA Purification Kit (Norgen, Thorold, ON, Canada). Retrotranscription of 200 ng of RNA was performed using the PrimeScript RT reagent kit (Takara Bio, Shiga, Japan). Finally, the cDNA obtained was amplified using TB Green Premix Ex Taq (Takara Bio, Japan). The primers used are detailed in Supplementary Table S1. The qPCR conditions were 30 s at 95 °C followed by 40 cycles of 5 s at 95 °C and 30 s at 60 °C. Each condition was performed in triplicate.

### 4.3. Microscopic Analysis of Mitophagy in Fibroblast Dermal Senescent Cells

Dermal fibroblasts were induced to a cellular senescent state as described in the previous section. Senescent fibroblasts were seeded in 35 mm culture dishes and treated for 48 h with hyaluronic acid plus succinic acid. Cells were then incubated with 100 nM of Mtphagy dye (Dojindo, Kumamoto, Japan) for 30 min. This reagent accumulates in mitochondria and emits red fluorescence only under acidic conditions, meaning that fluorescence increases when mitochondria are being degraded in the lysosome system. Thus, red fluorescence intensity correlates with mitophagic activity. Fluorescent images were taken for each condition in triplicate using the 3D Cell Explorer™ microscope (Nanolive, Tolochenaz, Switzerland). Fluorescence intensity per condition was quantified using ImageJ software (version 1.49) [31].

### 4.4. Adipogenesis Assay in Subcutaneous Adipocytes

Human subcutaneous preadipocytes were subcultured for 1–2 passages for low-passage control and 7–8 passages for high-passage conditions. High-passage conditions were used to produce aged cells simulating replicative aging after continuous subcultivation [32]. Cells were first cultured in preadipocyte differentiation medium (Promocell, Heidelberg, Germany) for 72 h. After differentiation induction, cells were incubated in preadipocyte nutrition medium (Promocell, Heidelberg, Germany) with or without 0.1% hyaluronic acid or 0.01% succinic acid for 17 days, changing the culture media every 2–3 days, according to the manufacturer’s protocol. Adipogenesis was then evaluated using Oil Red O staining kit, a red dye that specifically binds to intracellular fats. Cells were stained according to the manufacturer’s protocol (Merck Life Science, Darmstadt, Germany), and phase-contrast pictures were taken using a CKX41 phase contrast microscope (Olympus, Tokyo, Japan). Each condition was performed in triplicate. Oil Red O intensity was quantified using ImageJ as previously described [33].

### 4.5. Gene Expression Assay in Subcutaneous Adipocytes and Effect of Subcutaneous Adipocyte-Conditioned Medium Effect on Dermal Fibroblasts

Human subcutaneous preadipocytes were differentiated to adipocytes and incubated with hyaluronic acid and succinic acid as described in the previous section. At the end of the adipogenic induction protocol, cells were harvested and processed for qPCR gene expression analysis as described in the previous sections. The primers used are detailed in Supplementary Table S1. Each condition was performed in triplicate.

Before cell harvesting, conditioned medium from differentiated adipocytes treated with hyaluronic acid and succinic acid was transferred to human dermal fibroblasts, and the latter were incubated for 24 h to assess the effect of released factors by treated adipocytes on dermal fibroblasts. After 24 h, dermal fibroblasts were harvested and processed for qPCR gene expression analysis as described in the previous sections. The primers used are detailed in Supplementary Table S1. Each condition was performed in triplicate.

## 5. Conclusions

The present study showed that hyaluronic acid can induce autophagy-associated gene expression changes in senescent dermal fibroblasts, while succinic acid can positively affect the extracellular matrix and senescence-associated gene expression. Hyaluronic acid plus succinic acid significantly upregulated mitophagy in senescent dermal fibroblasts. On the other hand, hyaluronic acid and succinic acid regulated adipogenic gene expression in aged adipocytes while also inducing positive changes in dermal fibroblast gene expression after the treatment with conditioned medium from aged adipocytes that were previously treated with hyaluronic acid and succinic acid.

## Figures and Tables

**Table 1:** Gene expression of senescence and age-related genes in human dermal senescent fibroblasts treated with hyaluronic acid (HA) or succinic acid (Succ) after 48 h. Results are expressed as fold change compared to non-irradiated control fibroblasts. Standard deviation for each quantification is added in brackets, and statistical analysis is performed using Student’s *t* test, where * stands for *p* < 0.05, ** for *p* < 0.01, and *** for *p* < 0.001. Regarding statistical differences, UVB control cells are compared to no UVB control cells, and UVB irradiated cells treated with either hyaluronic acid or succinic acid were compared to UVB control cells.

	Gene	No UVB	UVB		
		Control	Control	HA	Succ
Extracellular matrix	*COL1A1*	1.00	1.26 (±0.11) *	1.36 (±0.04)	2.36 (±0.09) ***
	*CTGF*	1.00	1.49 (±0.27) *	1.62 (±0.23)	2.32 (±0.44) *
	*ELN*	1.00	1.26 (±0.09) *	1.34 (±0.05)	1.19 (±0.1)
	*FBN1*	1.00	1.64 (±0.1) **	1.4 (±0.15)	2.01 (±0.11) *
	*TGF-β1*	1.00	0.76 (±0.09) *	0.80 (±0.05)	0.88 (±0.08) *
	*LOXL1*	1.00	1.11 (±0.07)	1.10 (±0.05)	1.41 (±0.13) **
	*MMP-1*	1.00	2.22 (±0.13) **	2.02 (±0.17)	1.72 (±0.31) *
	*MMP-3*	1.00	1.60 (±0.11) **	1.16 (±0.03) *	0.84 (±0.07) **
	*MMP-14*	1.00	1.64 (±0.12) **	1.62 (±0.09)	1.62 (±0.12)
	*MME*	1.00	2.38 (±0.28) **	2.36 (±0.36)	2.35 (±0.24)
Senescence associated phenotype	*c-jun*	1.00	1.45 (±0.15) *	1.59 (±0.02)	1.51 (±0.04)
	*CXCL10*	1.00	1.79 (±0.14) **	1.56 (±0.2)	1.48 (±0.12) **
	*GDF15*	1.00	2.59 (±0.22) **	2.40 (±0.04)	2.41 (±0.10)
	*IL-6*	1.00	2.44 (±0.18) **	2.17 (±0.14)	1.23 (±0.08) **
	*IL-8*	1.00	3.00 (±0.36) **	3.78 (±0.41)*	2.06 (±0.55) *
	*BCL2*	1.00	1.57 (±0.25) *	1.80 (±0.12)	1.66 (±0.15)
Metabolism	*PGC1a*	1.00	1.45 (±0.12) *	1.43 (±0.06)	1.82 (±0.05) *
Autophagy	*ATG12*	1.00	1.54 (±0.16) *	1.89 (±0.14)	1.46 (±0.06)
	*ATG5*	1.00	1.24 (±0.02) **	1.50 (±0.04) **	1.20 (±0.09)
	*ATG7*	1.00	1.07 (±0.07)	1.77 (±0.04) **	1.43 (±0.05) *
	*Beclin1*	1.00	1.35 (±0.08) **	1.73 (±0.13) *	1.21 (±0.08)
	*LC3*	1.00	1.53 (±0.10) **	1.84 (±0.05) *	1.34 (±0.1)

**Table 2:** Gene expression of extracellular matrix and growth factors in human dermal fibroblasts treated with conditioned medium from hyaluronic acid plus succinic acid (HA + succ)-treated adipocytes. Results are expressed as fold change compared to non-irradiated control fibroblasts. Standard deviation for each quantification is added in brackets. Statistical analysis is performed using Student’s *t* test, where * stands for *p* < 0.05, ** for *p* < 0.01, and *** for *p* < 0.001. Regarding statistical differences, adipocyte-conditioned medium control is compared to control cells, and adipocyte-conditioned medium-treated cells with hyaluronic acid plus succinic acid are compared to adipocyte-conditioned medium control cells.

Gene	Control	Adipocyte-Conditioned Medium	
		Control	HA + Succ
*COL1A1*	1.00	1.07 (±0.05)	0.81 (±0.07)
*COL3A1*	1.00	1.70 (±0.11) **	1.92 (±0.08) **
*FN1*	1.00	1.60 (±0.25) *	3.30 (±0.24) *
*ELN*	1.00	0.54 (±0.1) ***	0.35 (±0.1)
*EGF*	1.00	0.42 (±0.04) **	1.17 (±0.06) **
*FGFB*	1.00	0.33 (±0.05) **	0.30 (±0.04)
*TGFB1*	1.00	0.49 (±0.04) ***	0.68 (±0.11)
*ACTA2*	1.00	1.39 (±0.17) *	1.83 (±0.16) *
*CCN1*	1.00	1.40 (±0.1) *	1.37 (±0.1)
*CEMIP*	1.00	0.61 (±0.05) **	0.60 (±0.07)

## Data Availability

Data is contained within the article and Supplementary Materials.

## Supplementary Materials

The following supporting information can be downloaded at https://www.mdpi.com/article/10.3390/ijms26157548/s1.
