# Supplementary material for: Hyaluronic and Succinic Acid: New Biostimulating Combination to Counteract Dermal and Subcutaneous Aging
Source: Int J Mol Sci. 2025 Aug 5;26(15):7548. doi: 10.3390/ijms26157548 (PMC12347182; doi:10.3390/ijms26157548)

Supplementary information

Supplementary Table S1. List of primers used for qPCR assay.

Gene	Forward sequence	Reverse sequence
COL1A1	5'-GATTCCCTGGACCTAAAGGTGC-3'	5'-AGCCTCTCCATCTTTGCCAGCA-3'
CTGF	5’-GGAAATGCTGTGAGGAGTGGGTGT-3´	5´-TGTCTTCCAGTCGGTAGGCAGCTA-3
ELN	5’-GCAGGAGTTAAGCCCAAGG-3'	5’-TGTAGGGCAGTCCATAGCCA-3'
FBN1	5’-GGTGAATGTACAAACACAGTCAGCAG-3'	5’-ATAGGAACAGAGCACAGCTTGTTGA-3'
TGFb1	5'-TGGCGATACCTCAGCAAC-3'	5'-ACCCGTTGATGTCCACTTG-3'
LOXL1	5'-AGCGCTATGCATGCACCTCTCATA-3'	5'-TGCAGAAACGTAGCGACCTGTGTA-3'
MMP1	5'-GGGAGATCATCGGGACAACTC-3'	5'-GGGCCTGGTTGAAAAGCA-3'
MMP3	5’-CCTGCTTTGTCCTTTGATGC-3'	5’-TGAGTCAATCCCTGGAAAGTC-3'
MMP14	5’-GCCTTCTGTTCCTGATAA-3'	5’-CCATCCTTCCTCTCGTAG-3'
MME	5’-TGCTGAGGGGTCACGATTTT-3'	5’-CTCAGTGGTGGCATCCATGT-3'
cJUN	5’-GACTGCAAAGATGGAAACGA-3'	5’-GGTCATGCTCTGTTTCAGGA-3'
CXCL10	5’-CCAGAATCGAAGGCCATCAA-3'	5’-CATTTCCTTGCTAACTGCTTTCAG-3'
GDF15	5’-TGGAGTCTTCGGAGTGCAAC-3'	5’-GCAAGAACTCAGGACGGTA-3'
IL6	5’-AGCCCTGAGAAAGGAGACATGTA-3'	5’-TCTGCCAGTGCCTCTTTGC-3'
IL8	5’-ATTTCTGCAGCTCTGTGTGAAGGT-3'	5’-TTTTTTATGAATTCTCAGCCCTCT-3'
BCL2	5’-ATGTGTGTGGAGAGCGTCAA-3'	5’-GCCGTACAGTTCCACAAAGG-3'
PGC1A	5’-TGAAGACGGATTGCCCTCATT -3’	5’-GCTGGTGCCAGTAAGAGCTT-3’
ATG12	5’-TAGAGCGAACACGAACCATCC-3’	5’-CACTGCCAAAACACTCATAGAGA-3’
ATG5	5’-GTTTTGGGCCATCAATCGGAA-3’	5’-TCTCCTAGTGTGTGCAACTGT-3’
ATG7	5’-ATGATCCCTGTAACTTAGCCCA-3’	5’-CACGGAAGCAAACAACTTCAAC-3’
Beclin1	5’-AGCTGCCGTTATACTGTTCTG-3’	5’-ACTGCCTCCTGTGTCTTCAATCTT-3’
LC3	5’-GATGTCCGACTTATTCGAGAGC-3’	5’-TTGAGCTGTAAGCGCCTTCTA-3’
IGF1	5’-CAGAGCAGATAGAGCCTGCG-3'	5’-CAGGTAACTCGTGCAGAGCA-3'
TNFA	5’-CGCTCCCCAAGAAGACAG-3'	5’-AGAGGCTGAGGAACAAGCAC-3'
COL3A1	5’-CTTCTCTCCAGCCGAGCTTC-3'	5’-TGTGTTTCGTGCAACCATCC-3'
FN1	5’-GTGCCTGATGTGGCCTTTTC-3’	5’- GACTCACACACCTATGGGCT-3’
EGF	5’-AATTGGTGGTGGATGCTGGT-3'	5’-GCTGCTGCAGTTTCCTTTCC-3'
FGFB	5’-CTGGCTATGAAGGAAGATGGA-3'	5’-TGCCCAGTTCGTTTCAGTG-3'
ACTA2	5’-CTTTGGCTTGGCTTGTCAGG-3'	5’-TCCCAGTTGGTGATGATGCC-3'
CCN1	5’-CGCCTTGTGAAAGAAACCCG-3'	5’-GGTTCGGGGGATTTCTTGGT-3'
CEMIP	5'- TGCTGCCCGGGTATTCAAAT-3'	5- 'CGTCCACTCCACGTCTTGAA-3'
p16	5’-GGCATTGTGAGCAACCACTG-3’	5’-CCTGTAGGACCTTCGGTGAC-3’

Supplementary Figure S1. Senescence-associated beta-galactosidase staining in non-irradiated control cells (A) and UVB-irradiated cells (B). (C) mRNA levels of senescence marker p16 (CDKN2A gene) in non-irradiated control cells and UVB-irradiated cells.

A B

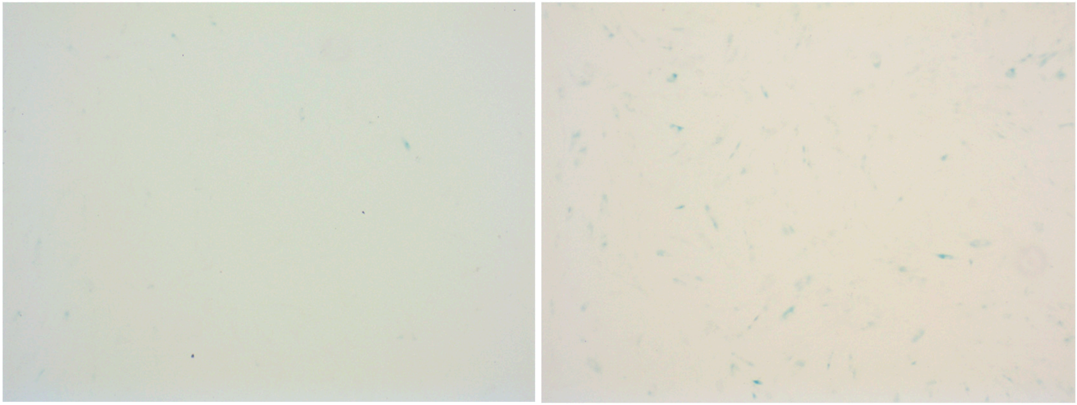

C

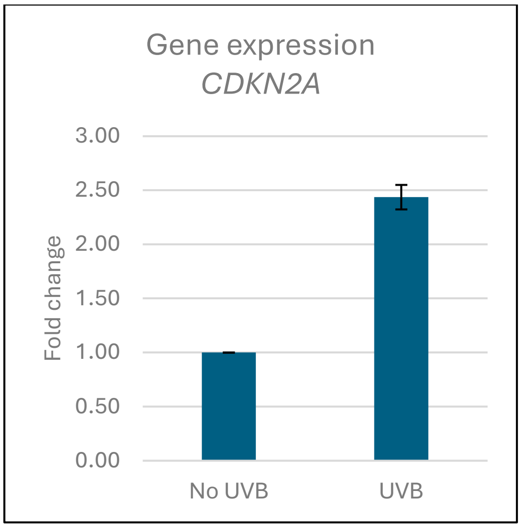

Supplement: Supplementary file 1 [file ijms-26-07548-s001.zip › ijms-3679913-supplementary.pdf]
